# CPDR: An R Package of Recommending Personalized Drugs for Cancer Patients by Reversing the Individual’s Disease-Related Signature

**DOI:** 10.3389/fphar.2022.904909

**Published:** 2022-06-20

**Authors:** Ruzhen Chen, Xun Wang, Xinru Deng, Lanhui Chen, Zhongyang Liu, Dong Li

**Affiliations:** State Key Laboratory of Proteomics, Beijing Proteome Research Center, National Center for Protein Sciences (Beijing), Beijing Institute of Lifeomics, Beijing, China

**Keywords:** precision oncology, personalized medicine, *in silico* prescription, Connectivity Map, drug repositioning

## Abstract

Due to cancer heterogeneity, only some patients can benefit from drug therapy. The personalized drug usage is important for improving the treatment response rate of cancer patients. The value of the transcriptome of patients has been recently demonstrated in guiding personalized drug use, and the Connectivity Map (CMAP) is a reliable computational approach for drug recommendation. However, there is still no personalized drug recommendation tool based on transcriptomic profiles of patients and CMAP. To fill this gap, here, we proposed such a feasible workflow and a user-friendly R package—Cancer-Personalized Drug Recommendation (CPDR). CPDR has three features. 1) It identifies the individual disease signature by using the patient subgroup with transcriptomic profiles similar to those of the input patient. 2) Transcriptomic profile purification is supported for the subgroup with high infiltration of non-cancerous cells. 3) It supports *in silico* drug efficacy assessment using drug sensitivity data on cancer cell lines. We demonstrated the workflow of CPDR with the aid of a colorectal cancer dataset from GEO and performed the *in silico* validation of drug efficacy. We further assessed the performance of CPDR by a pancreatic cancer dataset with clinical response to gemcitabine. The results showed that CPDR can recommend promising therapeutic agents for the individual patient. The CPDR R package is available at https://github.com/AllenSpike/CPDR.

## Introduction

Due to the multifaceted heterogeneity of cancer, the treatment response rate of patients is far below 100%. For example, a meta-analysis of phase II single-agent clinical studies shows that the median response rate for chemotherapy is only 11.9% and 30% for personalized targeted therapy (Schwaederle et al., 2015). Personalized drug use is important for improving the treatment response rate of cancer patients. The value of patient-derived transcriptomic data, which contain the key biological alterations triggering cancers (Casamassimi et al., 2017), have been recently demonstrated in guiding cancer patients’ personalized drug use (Rodon et al., 2019; Tuxen et al., 2019; Vaske et al., 2019).

The Connectivity Map (CMAP) is a hopeful computational approach for discovering personalized drugs based on patient-derived transcriptomic data. The CMAP measures the perturbed gene expression signatures of human tumor cell lines treated by various drugs, which are then compared with the signatures under a certain physiological or pathological condition, to reveal drug–gene condition associations (Lamb et al., 2006). Identifying potential anticancer drugs by associating perturbed signatures with cancer patients’ signatures is one of the successful applications of the CMAP (Sanda et al., 2010; Claerhout et al., 2011; Lim et al., 2014; Spijkers-Hagelstein et al., 2014).

Some anticancer drug recommendation tools have been developed on the basis of the CMAP, such as DrInsight (Chan et al., 2019), CMapBatch (Fortney et al., 2015), and OCTAD (Zeng et al., 2021). DrInsight can automatically create cancer signatures from a whole ranked gene list of differential analysis, avoiding the subjective impact during the cancer signature identification. CMapBatch is a meta-analysis tool, designed for applying the CMAP to multiple signatures of same cancer. Chen et al. (2017) established OCTAD, which supports the signature creation of cancer subtypes defined by molecular features before CMAP and proposed a method for drug effectiveness validation *in silico*. However, all these tools aimed at establishing associations between drugs and a bunch of cancer patients and are unable to give drug recommendation when coming to a single patient, which is a more common situation in clinical therapy compared to a large cohort of patient.

To fill this gap, here, we proposed a personalized drug recommendation tool based on CMAP and used an individual’s transcriptomic profile as the input. We developed a user-friendly R package—Cancer-Personalized Drug Recommendation (CPDR). CPDR consists of three steps: 1) Identification of an individual disease signature. 2) Candidate drug screening by reversing the individual disease signature. 3) *In silico* assessment of candidate drug efficacy. There are three features in CPDR: 1) Considering the widespread and stochastic biological alterations unrelated to the disease status in an individual’s transcriptomic profile, CPDR identifies the disease signature by using a patient subgroup as biological replicates, which have phenotypes and transcriptomic profiles similar to those of the individual. 2) For the subgroup with high infiltration of non-cancerous cells, CPDR supports profile purification to extract gene expression patterns of cancer cells. 3) CPDR supports *in silico* drug efficacy assessment using drug sensitivity data of cancer cell lines. In *Results and Discussion* section, we demonstrated the workflow of CPDR with the aid of a colorectal cancer dataset and performed the *in silico* validation. We further verified the effectiveness of CPDR using a pancreatic cancer dataset with clinical response to gemcitabine.

## Materials and Methods

### Background Data and Data Preprocessing

CPDR used gene expression profiles of cancer patient cohorts from The Cancer Genome Atlas project (TCGA) and those of human normal tissues from The Genotype-Tissue Expression Database (GTEX) (Cancer Genome Atlas Research et al., 2013; Consortium, 2013). For the cancer patient cohorts, molecular information and RNA-seq count data were downloaded by using the R package ‘cBioPortal’ (Cerami et al., 2012), and for human normal tissues, RNA-seq count data were downloaded by using the R package ‘OCTAD’ (Zeng et al., 2021).

The drug perturbation data were downloaded from the Library of Integrated Network-Based Cellular Signatures (LINCS, Level 5, Accession Number: GSE70138). LINCS is an expanded project of CMAP, which contains perturbed signatures for 1,808 compounds at a variety of durations, concentrations, and cell lines (Subramanian et al., 2017). The 10,174 ‘best-inferred genes’ with high fidelity were used as drug perturbation signatures in CPDR.

In order to perform *in silico* validation, CPDR also integrated baseline (*i.e.,* pre-treatment) gene expression profiles and drug sensitivity data on cancer cell lines. The former were obtained from the Cancer Cell Line Encyclopedia project (CCLE), involving baseline gene expression profiles of 1,036 cell lines covering 36 cancer types (Barretina et al., 2012). The latter were downloaded from the Profiling Relative Inhibition Simultaneously in Mixtures project (PRISM), involving drug sensitivity data from 499 cell lines treated by 1,448 compounds (Yu et al., 2016). We further unified drug names using the PubChem online tool (Kim et al., 2021) and unified cell line names using the R package ‘PharmacoGx’ (Smirnov et al., 2016). Finally, we obtained a total of 661 consensus drugs and 475 consensus cell lines between PRISM and CCLE.

### Data for the Use Case

(1) Colorectal cancer dataset: to demonstrate the workflow of CPDR, in the use case Ⅰ, we used a colorectal cancer dataset as an example. This dataset was obtained from the GEO database (GSE164541), containing gene expression profiles of five colorectal cancer patients.
(2) Pancreatic cancer dataset: to further assess the performance of CPDR, in the use case Ⅱ, we applied CPDR on a pancreatic cancer dataset. This dataset was obtained from the CTR-DB (dataset ID in CTR-DB: CTR_RNAseq_202), containing baseline gene expression profiles of 46 patients with known treatment response to gemcitabine. CTR-DB has comprehensively collected and uniformly reprocessed 83 patient-derived clinical transcriptome source datasets with cancer drug response (involving 28 cancer types and 123 drugs) and meanwhile provided various analysis functions facilitating the integration and re-mining of these data (Liu et al., 2022).

### Mechanisms of CMAP

Most cancer mutations are passengers, which makes it difficult to find driver mutations for an individual patient. In addition, a study focusing on genome-driven oncology concluded that only 7% (63 out of 843) of tumor patients who received molecular screening could benefit from targeted therapy (Massard et al., 2017), largely as a consequence of the low coverage of existing targetable driver mutations. It has been revealed that the widespread molecular variability is often reduced to a much smaller set of pathway-based dysfunctions (Hanahan and Weinberg, 2011; Vogelstein et al., 2013; Menche et al., 2017). This research paradigm at the system level offered a new opportunity for personalized therapy. Therefore, we considered CMAP as a hopeful approach.

CMAP measures the therapeutic effect of a drug on disease at the transcriptomic level. To be more specific, CMAP conducted treatment experiments in various human tumor cell lines with various drugs at diverse concentrations and durations and then collected paired gene expression profiles (control and treatment). The fold change values of treatment verse control were calculated and converted to rank values, which constitute the so-called perturbed signatures representing the pattern of action of the corresponding treatment. The disease signatures, which are usually from the differential analysis between the disease and normal samples, represent the pattern of action of a specific disease state.

A comparison between disease and perturbed signatures allows the discovery of therapeutic drugs. Notably, to reduce false discoveries due to the lack of statistical control in perturbed signatures caused by a few paired profiles, CMAP adopts a rank-based and pattern-matching strategy. As shown in Figure 2, the disease signature is split into an upregulated set and a downregulated set. Then, two Kolmogorov–Smirnov (KS) statistics are calculated, respectively (a and b), which mean positive and negative concordances between the regulated sets and each perturbed signature. If the positive concordance value is greater than the negative one, it is retained as a well-matched result. If the negative concordance value is greater than the positive one, the negative one with a minus sign is retained as a reversely matched result. Finally, a connectivity score is assigned for measuring the comprehensive concordance between the regulated sets (*i.e.* the disease signature) and the perturbed signature. A positive connectivity score denotes the drug-induced effect is similar to the disease effect. In contrast, a negative concordance denotes the reversal effect, which indicates the drug is a potential therapeutic agent.

### Identification of the Individual Disease Signature

Considering the widespread and stochastic biological alterations unrelated to the disease status in an individual’s transcriptome, we used the disease signature obtained from the subgroup with transcriptomic profiles similar to those of the input patient. Given the gene expression profile of an individual cancer patient, its individual disease signature was obtained by four steps as follows.

(1) Recognition of the cancer subtype that the input patient belongs to. First, we identified the cancer subtypes based on the TCGA patient cohort with the same cancer type as that of the input patient. After a log2 transformation of expression profiles, we extracted the 1,500 most variant genes across samples for the following unsupervised consensus clustering. Then, the non-negative matrix factorization (NMF) method was used for the clustering (Brunet et al., 2004). We determined the optimal clustering/subtyping result by considering the cophenetic scores and average silhouette widths of different solutions (Brunet et al., 2004; Xu et al., 2017). We defined the similarity value between the input patient and each cancer subtype as the median value of the Spearman rank correlation coefficients between the input patient and the ones in the cancer subtype, computed based on the 1,500 most variant genes. Finally, the subtype with the maximal similarity value was considered as the one the input patient belongs to.
(2) Identification of the subgroup that the input patient belongs to. For acquiring a closer cohort from the corresponding subtype as the biological replicates of the input patient, we further identified the subgroup the patient belongs to. The subtype samples with Spearman rank correlation coefficients (calculated in step 1) ranked in the top N were considered as the subgroup the input patient belongs to. Referring to a survey of statistical power to detect differentially expressed genes (DEGs) (Conesa et al., 2016), we assumed 3, 5, and 10 to be the optional sizes of the biological replicates/subgroup in CPDR.
(3) Identification of the individual disease signature. Considering few paired non-tumor samples in TCGA posing a challenge to differential gene expression analysis, the GTEX database was determined to be the source of normal samples. However, first, the experimental processing of GTEX is different from TCGA, which can lead to batch effects (Wang et al., 2018; Arora et al., 2020). Second, it is crucial for differential analysis to select biologically sound control. To make data from different sources more compatible, the UCSC Xena project (Caicedo et al., 2020) has recomputed raw RNA-seq data based on a standard pipeline. To choose biologically sound control, Zeng et al. developed an auto-encoder to extract features for each sample from UCSC-derived profiles. The t-SNE plot and similarity measurement based on encoded features showed that the batch effect among different databases was minimized, and GTEX normal samples highly correlated with TCGA tumor samples to tend to have same or similar tissue origins (Zeng et al., 2019). Therefore, in CPDR, we used the UCSC-harmonized data to compute differential expression genes and also used the encoded features from OCTAD to select biologically sound control for the subgroup the input patient belongs to. The obtained DEGs constituted the individual disease signature. In addition, we provided a batch correction option (normalize_samples) that uses RUVSeq (Risso et al., 2014) to minimize batch effects when users perform differential analysis with DESeq (Love et al., 2014) or edgeR (Robinson et al., 2010). We also provided limma voom (Law et al., 2014) for differential analysis which is used by UCSC Xena and GEPIA (Tang et al., 2017).

### Purification of Gene Expression Profiles

The bulk tumors (i.e., the patient samples) comprise populations of different cell types (Liotta and Petricoin, 2000). Thus, the gene expression pattern of cancer cells could be blurred by non-cancerous cells (Bachtiary et al., 2006). CPDR supports tumor microenvironment (TME) analysis, and for subgroups with high non-cancerous cell infiltration, CPDR supports gene expression profile purification before the identification of the disease signature.

(1) TME analysis: the aim of this analysis was to explore the extent of non-cancerous cell infiltration of patient samples. Based on sample gene expression profiles, we performed the single-sample gene set enrichment analysis (ssGSEA) using the signature genes defined by the R package ‘estimate’ to infer the fraction of stromal and immune cells and using the signature genes defined by Pornpimol Charoentong et al. (2017) to predict the abundance of 28 immune cell populations.
(2) Gene expression profile purification: for subgroups with high non-cancerous cell infiltration, the gene expression profile purification was performed with the aid of ISOpure (Anghel et al., 2015), which is a deconvolution method to directly extract the expression pattern specific to cancer cells from the heterogeneous tumor bulk (Shen-Orr and Gaujoux, 2013).

### Candidate Drug Screening by Reversing the Disease Signature

Previous studies have shown that there is time and dose dependence in LINCS, that is, treatments under long duration and high concentration are more likely to disturb genes (Lim and Pavlidis, 2021). To obtain the unbiased estimation of drug reversal efficacy, we used the summary reverse gene expression score (sRGES) proposed by Chen et al. (2017) to measure the effect of drugs on the reversal of the individual disease signature. sRGES is a linear combination of connectivity scores across different treatments. For any drug in LINCS, the standard treatment (10 μM concentration and 24 h duration) is set as the reference, and any other treatment was set as the target. A reward function is used to standardize the connectivity score for the target treatments. Ultimately, after the simple linear combination of standardized connectivity scores, one sRGES is assigned to each drug.

### *In silico* Estimation of Candidate Drugs

CPDR supports *in silico* validation of predicted candidate drugs by three steps as follows.

(1) Recognition of the input patient-relevant cell line: we selected a CCLE cancer cell line most relevant to the input patient based on the gene expression profile similarity. We computed the gene expression profile similarity based on 1,500 most variant genes across all CCLE cell lines. The similarity was measured by the Spearman rank correlation coefficient between a cell line and the input patient. The cell line with the highest correlation coefficient was considered to be the input patient-relevant cell line.
(2) Defining effective and ineffective drugs: we used the area under the drug dose response curve (AUC) to measure the drug efficacy on a cell line. For each cell line, effective drugs were defined as those with AUCs at least 0.5 standard deviation (SD) less than the mean, and other drugs were ineffective drugs.
(3) *In silico* evaluation of drug effectiveness

Here, we used three methods to perform the evaluation.

(a) Calculating the correlation between sRGES scores and drug efficacy AUCs.

We used the *in silico* evaluation method of drug effectiveness proposed by Chen et al. (2017). For an individual, a high Pearson correlation coefficient, between sRGES of the predicted drugs for the patient and drug efficacy AUCs on the patient-relevant cell line, means a good prediction performance.

(b) Calculating the sRGES difference between the effective and the ineffective drugs on the individual-relevant cell line by *t*-test.
(c) Comparing with the null distribution: we randomly permutated the relationship between the individual and the subgroup it belongs to. For each random, the Pearson correlation coefficient between sRGESs and drug efficacy AUCs was computed. The random process was repeated 100 times, constituting the null distribution. We used the one sample *t*-test to determine the statistical significance of the drug prediction result.

## Results and Discussion

Considering CMAP is a hopeful computational approach for personalized drug recommendation, we proposed a novel tool named Cancer-Personalized Drug Recommendation (CPDR), which is designed for personalized drug recommendation based on CMAP and using an individual’s transcriptomic profile as the input.

### Function Descriptions and Principles

**FIGURE 1:**
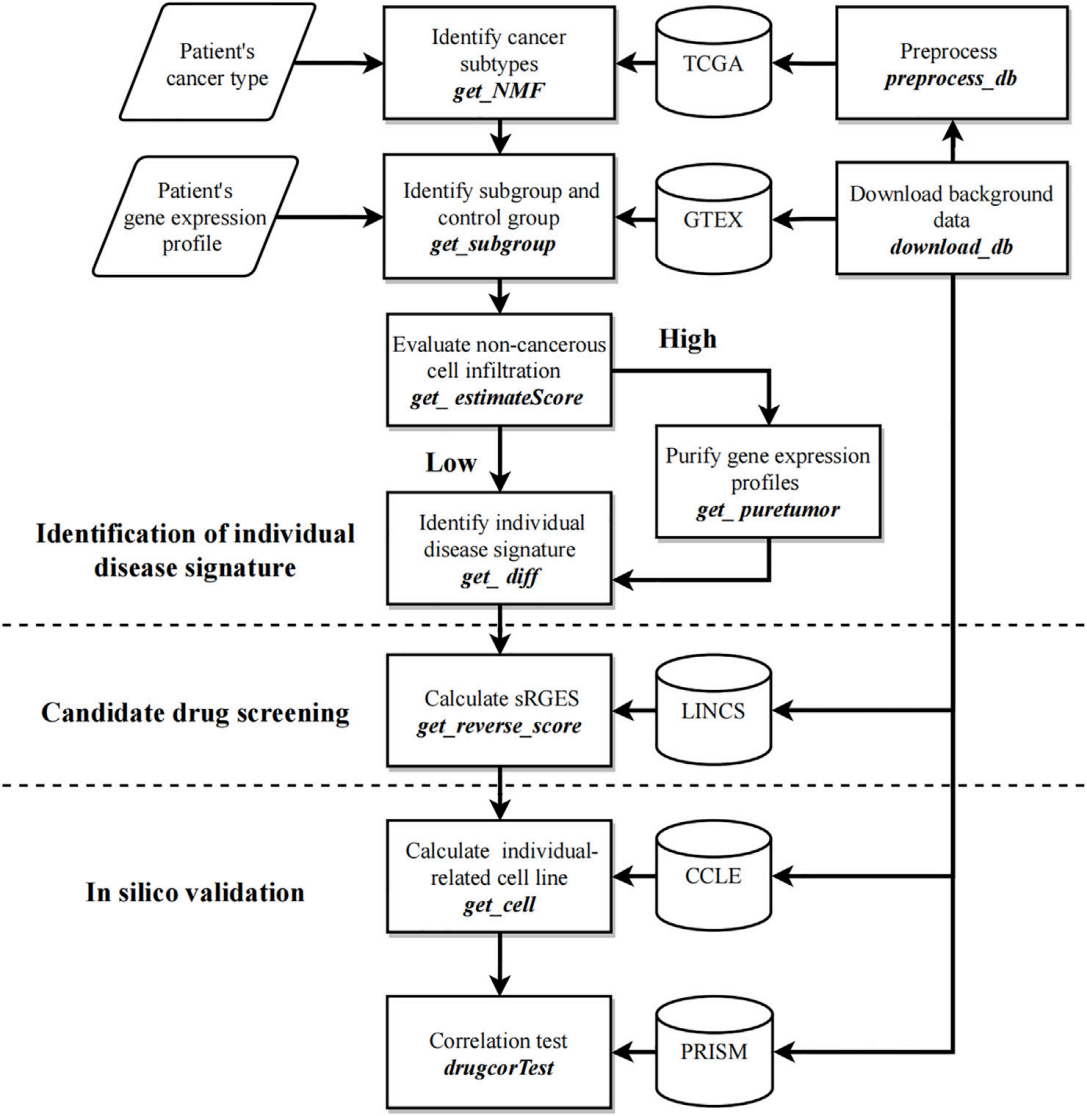
Workflow of CPDR. The flow diagram shows the user’s inputs (parallelogram), the functions (rectangles), and the background data (cylinder). CPDR has two data preparing functions to download and preprocess background data (right functions) and eight analysis functions for identifying an individual disease signature, screening, and validating personalized drugs (left annotations).

Figure 1 shows the workflow of CPDR. The inputs are the gene expression profile of an individual patient, the corresponding cancer type provided by the user, and the background data downloaded by two CPDR data preparation functions. In addition, CPDR has eight analysis functions for identifying individual disease signature, screening, and validating personalized drugs. All these functions are introduced as follows.

### Data Preparation Functions

*CPDR:download_db* implements background data download, including the following:

(1) TCGA cohorts with 32 optional cancer types. Given the cancer type of the input patient (setting parameter *tset*), a MultiAssayExperiment object will be downloaded from cBioPortal, which contains RNA-seq count data of patient samples for that cancer type. It will be saved in the ‘CPDR_db/TCGA’ directory by default.
(2) GTEX datasets (including 51 healthy tissues). Setting parameter *nset = ‘GTEX’*, a file named ‘octad.counts.and.tpm.h5’ will be downloaded and saved in the ‘CPDR_db/GTEX’ directory by default.
(3) CCLE, PRISM, and LINCS are necessary pharmacogenomic datasets (see Methods) which can be downloaded by setting *pset = c(‘CCLE’*, *‘PRISM’*, *‘LINCS’)* and will be saved in the ‘CPDR_db/Pharmacogenomic’ directory by default.

*CPDR::select_db* implements data preprocessing, including unifying gene names, and removing batch effects. The inputs are the downloaded TCGA cohort and RNA-seq count data of the input patient. Since the previous study has shown the weak correlation between the RNA-seq and microarray data for the same biological sample (Buzdin et al., 2014), we suggested not to input microarray data here. It unifies gene names into gene symbols with the aid of R package ‘clusterProfiler’ (Wu et al., 2021), and only shared genes between TCGA cohort and the input patient are retained. Finally, it uses R package ‘limma’ to remove batch effects between them and outputs the preprocessed gene expression profiles.

### Analysis Functions

We identified the individual disease signature by using the subgroup with transcriptomic profiles similar to those of the input patient. Specifically, first, we recognized the cancer subtype which the input patient belongs to. Then, we extracted a precise patient group (*i.e.*, subgroup) within this subtype as the biological replicates of the input patient. Finally, the gene expression profiles of the subgroup and the corresponding control group were used for the differential gene expression analysis. The differentially expressed genes (DEGs) consist of the individual disease signature of the input patient.

*CPDR:get_NMF* implements the recognition of cancer subtypes by the NMF method. The inputs are the preprocessed gene expression profiles of TCGA cohort, and the output is the cancer subtyping result (Figure 3A).

*CPDR::get_subgroup* implements the identification of the subgroup and the control group. The inputs are the cancer subtyping result and the preprocessed gene expression profile of the input patient, and the output is the normalized RNA-seq count data of the subgroup and the corresponding control group (see Methods) (Figure 3B).

*CPDR::get_estimateScore* implements the evaluation of non-cancerous cell infiltration of each cancer subtype (see Methods). The inputs are the cancer subtyping result and the preprocessed gene expression profiles of TCGA cohort, and the output is an infiltration score matrix of non-cancerous cells (Figure 3C).

*CPDR::get_puretumor* implements profile purification of the subgroup with a high non-cancerous infiltration score by the ISOpure method (see Methods). This function is aimed at extracting the expression pattern of cancer cells to improve the prediction performance of the CMAP. The input is the normalized RNA-seq count data of the subgroup and the control group, and the outputs are purified gene expression profiles.

*CPDR::get_diff* implements the identification of the individual disease signature by differential gene expression analysis. The input is the normalized RNA-seq count data of the subgroup and the control group, and the outputs are DEGs. CPDR provides three differential analysis methods (limma, DEseq, and edgeR), and a batch normalization method (RUVseq).

*CPDR::get_reverse_score* implements the calculation of the Summary Reverse Gene Expression Score (sRGES), which evaluates the reversal efficacy of drugs on the input individual disease signature (see *Methods*). The output is a candidate drug list with sRGES scores, and the lower value of sRGES means a stronger reversal efficacy.

*CPDR::getcell* implements the recognition of the individual-relevant cell line based on RNA-seq count data on the input patient (see Methods) and used for the *in silico* validation of drug effectiveness at the cell line level.

*CPDR::drugcorTest* implements the *in silico* validation. The results include the Pearson correlation coefficients between sRGES scores of the predicted drugs and their drug efficacy AUCs on the patient-relevant cell line and the sRGES difference between effective and ineffective drugs (see *Methods*) (Figure 3D).

### Use Case I: Personalized Drug Recommendation for Colorectal Cancer Patient PT1

Colorectal cancer is the third most common cancer worldwide and the second most common cause of cancer-relevant death (Global Burden of Disease Cancer et al., 2017). Currently, the main chemotherapy for colorectal cancer is fluoropyrimidine (5-FU), with response rates only at 10–15% (Kelly and Goldberg, 2005). It is important to develop tailored treatments for colorectal cancer patients. Here, we demonstrated the workflow of CPDR using a colorectal cancer dataset as an example. This dataset (GSE164541) contains gene expression profiles of the primary tumors from five patients, and we tried to find personalized therapeutic agents for them using CPDR.

### Patients Belonging to S-3 and S-4 Subtypes

Guinney et al. (2015) developed a consensus molecular subtype (CMS) system for colorectal cancer, which has clear functional characteristics for each subtype. Considering the CMS system that has demonstrated clinical utility in predicting patient therapy response (Mooi et al., 2018; Lenz et al., 2019), we employed it as the standard to detect the reliability of our subtyping result. The colorectal cancer patient cohort from TCGA was downloaded by *CPDR::download_db* (*tset = ‘coadread’*) and preprocessed by *CPDR::select_db* (i.e., unifying gene names, removing batch effects).

**FIGURE 2:**
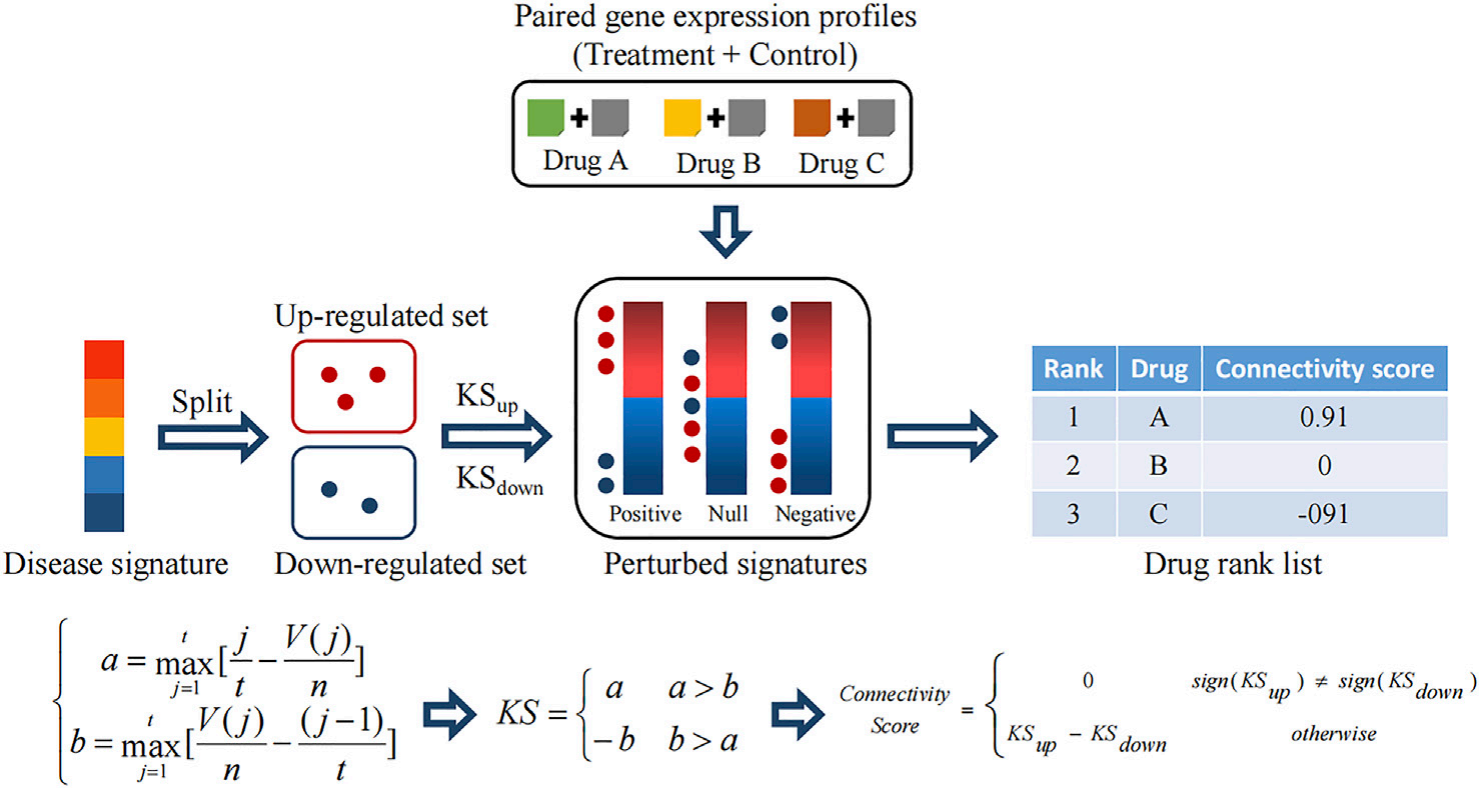
Mechanistic overview of the Connectivity Map (CMAP).

**FIGURE 3:**
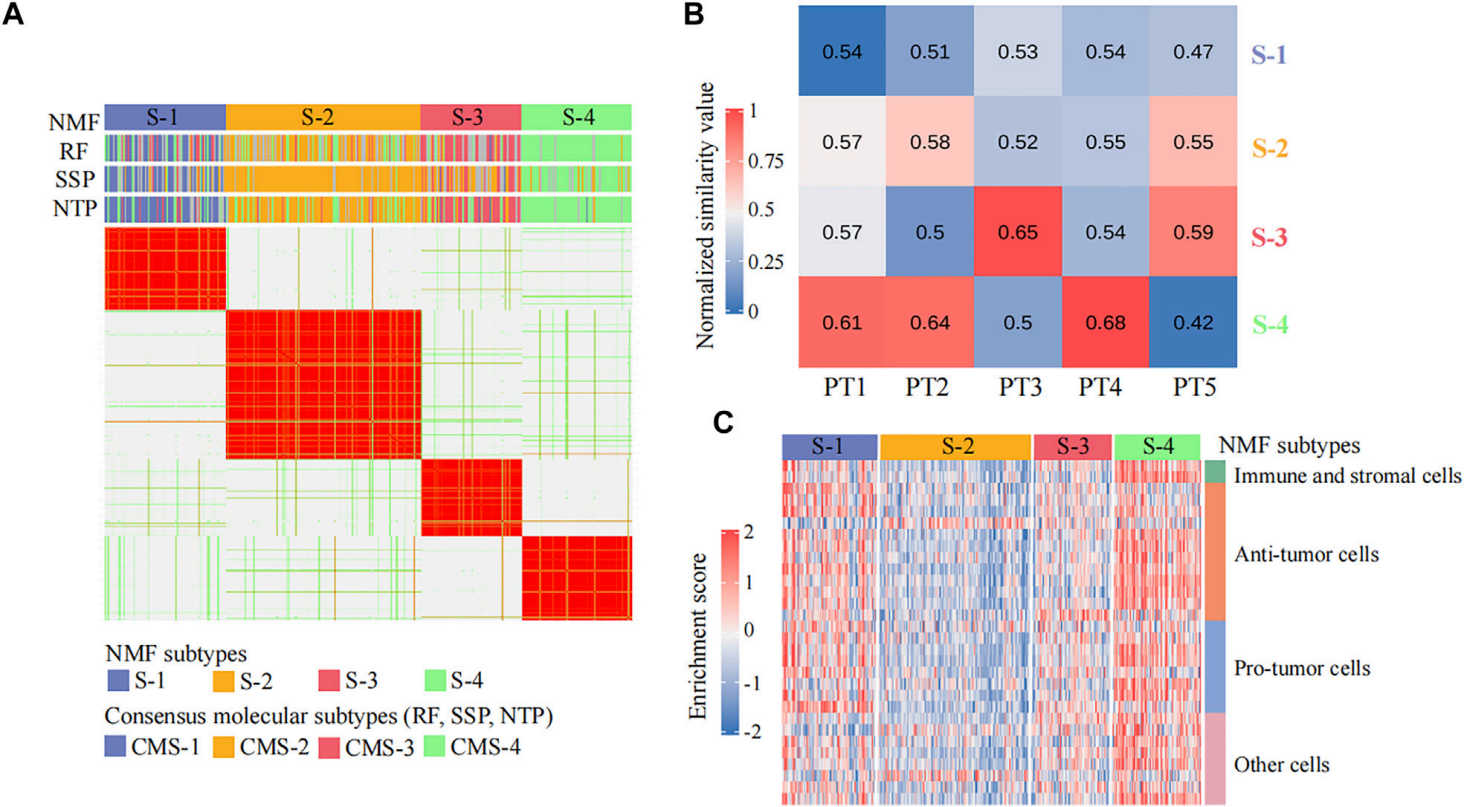
Identification of individual disease signatures for five colorectal cancer patients. **(A)** Heatmap of the NMF consensus matrix of 365 TCGA colorectal cancer patient samples. Rows and columns are samples, and the similarity between samples is colored in the body of the heatmap. The column annotation bars show four clustering methods: the non-negative matrix factorization (NMF), the random forest (RF), the single-sample predictor (SSP), and the nearest template prediction (NTP), respectively. The NMF method is used by CPDR, and others are CMS classifiers from R package ‘CMScaller’ and ‘CMSclassifier.’ **(B)** Heatmap of similarity values between five colorectal cancer patients and four NMF subtypes. Each column represents an input patient, and each row represents a cancer subtype. The value in each cell is the median value of the Spearman rank correlation coefficients between the input patient and the ones in the cancer subtype, computed across the 1,500 most-variant genes (see Methods). **(C)** Heatmap of the non-cancerous cell infiltration score matrix. The row annotation bar represents four types of non-cancerous cells, and the column annotation bar represents four NMF subtypes. The enrichment scores computed by ssGSEA are colored in the body of the heatmap.

A total of 365 colorectal cancer patient samples were classified into four subtypes (sample size: S-1 = 84, S-2 = 135, S-3 = 70, and S-4 = 76) by *CPDR::get_NMF* (Figure 2A; Supplementary Figures 1A, B). Then, we compared our subtyping result with the CMS system defined by CMS classifiers from the R package ‘CMScaller’ and ‘CMSclassifier.’ The Pearson correlation test result showed our subtyping result was highly correlated with the CMS system (the average correlation coefficient is 0.75) (Figure 3A), and the Fisher exact test and gene set enrichment analysis results revealed their clear correspondence: S-1, S-2, S-3, and S-4 correspond to CMS-1, CMS-2, CMS-3, and CMS-4, respectively (Supplementary Figures 1C,D). Finally, we determined the cancer subtypes for the five input patients by *CPDR::get_subgroup*. PT1, PT2, and PT4 belonged to S-4, that is, CMS-4, while PT3 and PT5 belonged to S-3, that is, CMS-3 (Figure 3B).

### Identification of Individual Disease Signatures From Purified Gene Expression Profiles

We further used *CPDR::get_subgroup* to identify subgroups to which each of the five patients belonged and also identified the corresponding normal control groups with the same size. Next, we assessed the non-cancerous cell infiltration scores of each subgroup using the *CPDR::get_estimateScore*. From Figure 3C, we found that the S-4 where the patients PT1, PT2, and PT4 belonged was high infiltration and therefore required gene expression profile purification using *CPDR::get_puretumor*. Furthermore, differential gene expression analysis was performed using *CPDR::get_diff* to obtain the individual disease signatures. Comparing the amount of DEGs obtained before and after purification, we found that differential analysis using purified tumors identified more DEGs (Supplementary Figure S2), which is consistent with the previous report (Quon et al., 2013).

### Reliable Prediction of Personalized Candidate Agents

**FIGURE 4:**
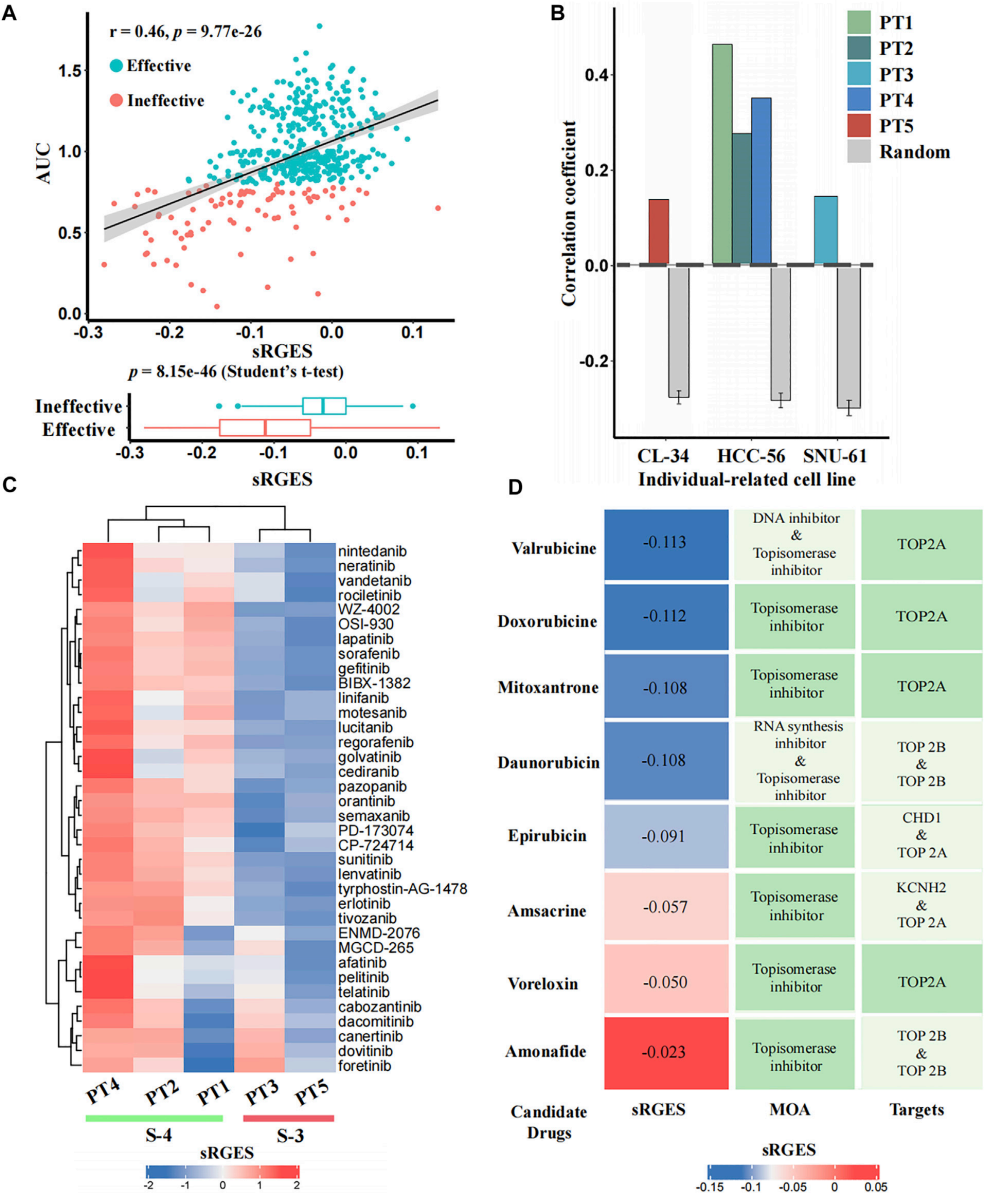
*In silico* estimation of the effectiveness of predicted drugs. **(A)** Correlation analysis between sRGES scores and efficacy AUCs of the predicted drugs (the top panel) and differential analysis (*t*-test) of sRGES scores between effective and ineffective drugs (the bottom panel) on PT1-relevant cell line. The *y*-axis of the top panel represents the median AUC of multiple treatments of a drug on this cell line. See Supplementary Figure S3 for results of other four patients. **(B)** Comparison results of the correlation coefficients between five patients and null distributions. The *x*-axis represents the individual-related cell lines of the five patients, and the *y*-axis represents the Pearson correlation coefficients between sRGES scores and drug efficacy AUCs. **(C)** sRGES scores of EGFR and VEGFR inhibitors of five patients. **(D)** Predicted topoisomerase inhibitors for PT1 and the corresponding sRGES scores, MOA, and target annotations.

To identify candidate drugs that can reverse the individual disease signature, we calculated the sRGES scores of 661 drugs for each input patient using *CPDR::get_reverse_score*. To assess prediction efficacy, we further used CCLE cell lines to simulate patients (*Methods*), and calculated the Pearson correlation coefficients between drug efficacy AUCs of the patient-related cell line and the sRGES scores of predicted drugs. In total, we identified three individual-related cell lines for five patients. PT1, PT2, and PT4 were simulated by HCC-56, PT3 by SNU-61, and PT5 by CL-34. For all five input patients, the sRGES of predicted drugs were significantly and positively correlated with the drug efficacy AUCs on the corresponding individual-related cell lines (Pearson correlation coefficients were 0.46, 0.28, 0.15, 0.35, and 0.14, and *p*-values were 9.77e-26, 1.67e-09, 1.41e-03, 9.46e-15, and 3.82e-03, respectively) (see Figure 4A for PT1 and Supplementary Figure S3 for other patients), and the coefficients were all significantly higher than those of the random null distribution (one sample *t*-test, *p*-values were 2.25e-71, 1.20e-59, 2.85e-48, 1.10e-64, and 5.18e-25, respectively) (Figure 4B). In addition, we also compared sRGES scores between the effective and ineffective compounds of the relevant cell lines and found the difference was statistically significant (*t*-test, *p*-values were 8.51e-46, 1.70e-09, 1.40e-03, 1.00e-14, and 3.80e-03, respectively) (see Figure 4A for PT1 and Supplementary Figure S3 for the other patients). These *in silico* estimation results indicated the reliability of predicted candidate drugs, to some extent.

Previous studies have documented that CMS-3 is sensitive to EGFR and VEGFR inhibitors, while CMS-4 is resistant to them (Sawayama et al., 2020). The sRGES of 36 known EGFR and VEGFR inhibitors from the PRISM database showed that PT3 and PT5, which belong to the CMS-3, had lower sRGES scores than PT1, PT2, and PT4, which belong to CMS-4 (Figure 4C). This further demonstrated the reliability of our prediction.

### Doxorubicin and Valrubicin Are Recommended Drugs for PT1

We used PT1 as an example for which we tried to further identify effective drugs simultaneously based on prediction results and prior knowledge. Previously, topoisomerase inhibitors targeting TOP2A were reported to be sensitive drugs for CMS-4 that PT1 belongs to (Sveen et al., 2018; Carvalho et al., 2021; Fohlen et al., 2021), and we did find that PT1 showed parent sensitivity (sRGES < -0.1) to four of the eight topoisomerase inhibitors targeting TOP2A (mean sRGES was -0.11, and the mean rank was 78) (Figure 4D). We used the PT1-related cell line (HCC-56) to further filter effective drugs. By setting sRGES < -0.1 and drug category as effective, only drugs with low sRGES and effective on HCC-56 were retained. We ultimately recommended two compounds of four candidates for PT1: doxorubicin and valrubicin.

### Use Case II: Estimation on Clinical Patients With Drug Response

**FIGURE 5:**
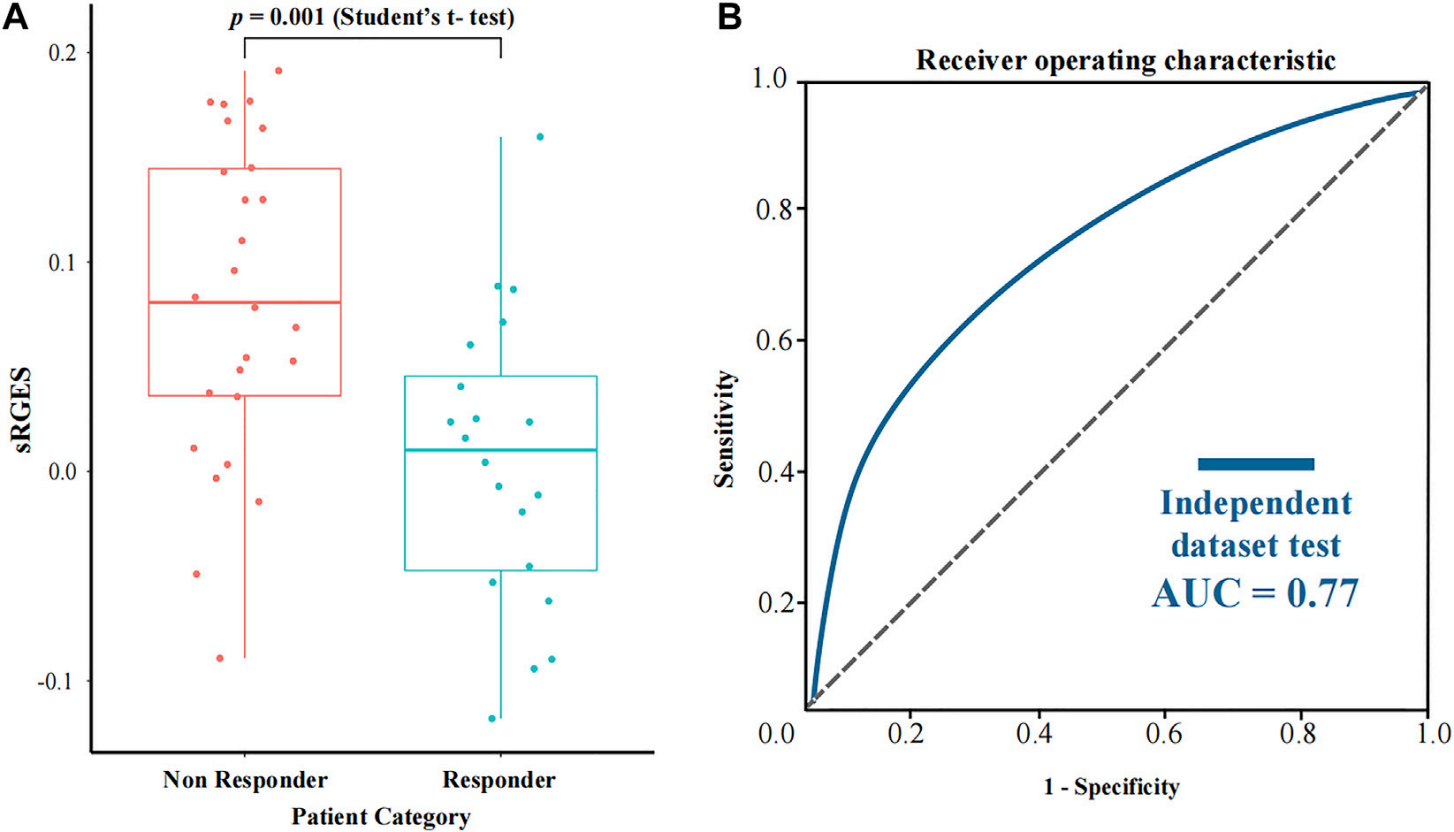
Assessment results of CPDR on a pancreatic cancer dataset with clinical response to gemcitabine. **(A)** Differential analysis of sRGES scores between response and non-response groups by *t*-test. **(B)** ROC curve and AUC of the sRGES score of gemcitabine-discriminating responders and non-responders.

In order to further assess the performance of CPDR, we used a pancreatic cancer dataset downloaded from the CTR-DB, which has baseline RNA-seq profiles of 46 patients and their response to gemcitabine. Considering that resistance is influenced by concentration and duration of treatment, we uniformly used the standard treatment (10 μM concentration and 24 h duration) of gemcitabine to compute sRGES for each patient, then stratified patients according to actual clinical outcomes (defined as resistant or sensitive to gemcitabine), and compared predicted sRGES scores between the two groups by *t*-test (Figure 5). The results showed that the predicted sRGES scores of gemcitabine were able to correctly classify patients into the responder/non-responder category (*t*-test, *p*-value = 0.001) with an AUC of 0.77. This further validated the effectiveness of CPDR.

## Data Availability

The original contributions presented in the study are included in the article/Supplementary Material; further inquiries can be directed to the corresponding authors.
