# Supplementary material for: CPDR: An R Package of Recommending Personalized Drugs for Cancer Patients by Reversing the Individual’s Disease-Related Signature
Source: Front Pharmacol. 2022 Jun 20;13:904909. doi: 10.3389/fphar.2022.904909 (PMC9252520; doi:10.3389/fphar.2022.904909)
Supplement: Supplementary file 1 [file DataSheet1.PDF]

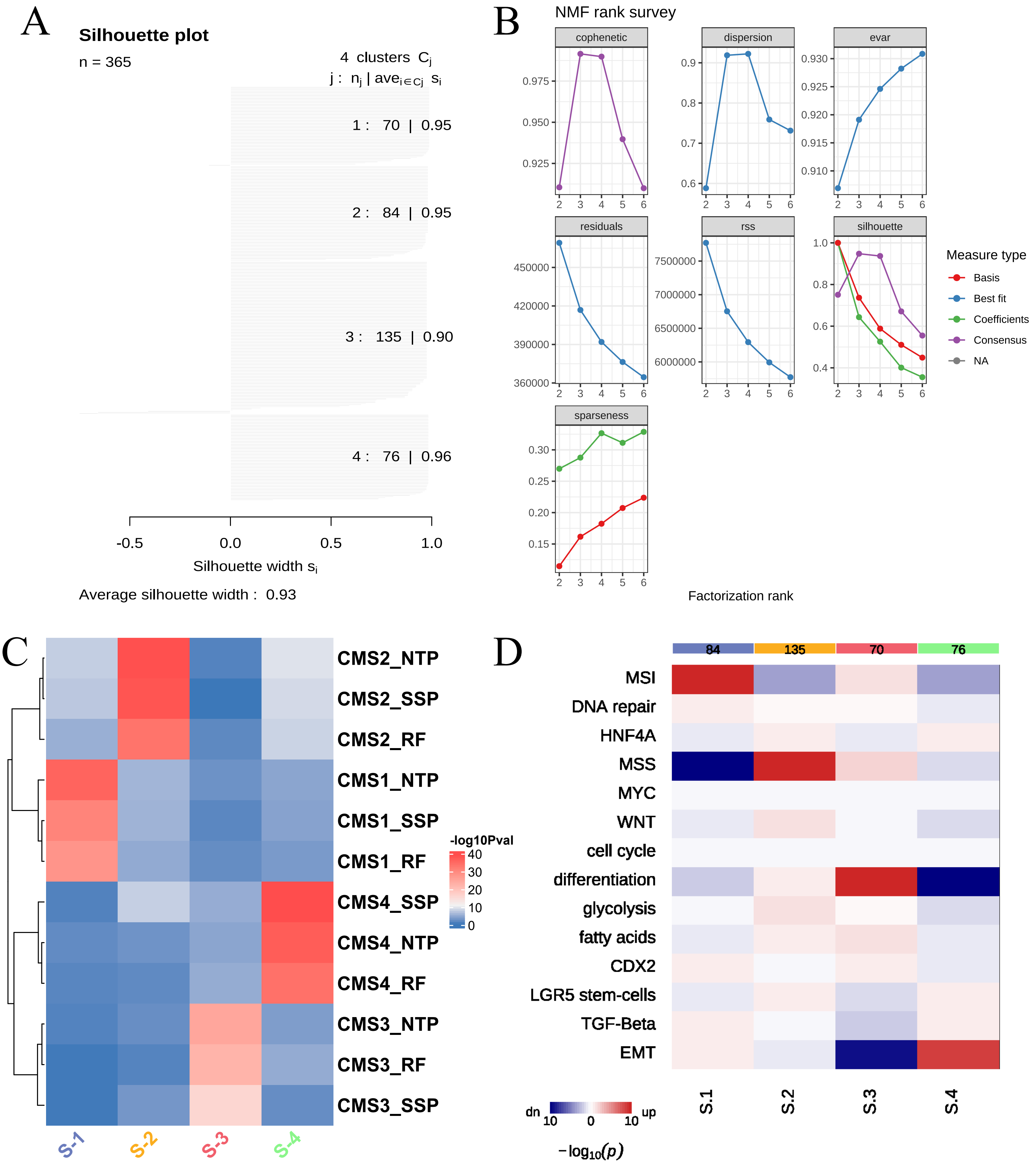
A
Silhouette plot
n = 365
4 clusters C_j
j : n_j | $ave_{i \in C_j}$ s_i
1 : 70 | 0.95
2 : 84 | 0.95
3 : 135 | 0.90
4 : 76 | 0.96
-0.5
0.0
0.5
1.0
Silhouette width s_i
Average silhouette width : 0.93
B
NMF rank survey
cophenetic
dispersion
evar
residuals
rss
silhouette
sparseness
Measure type
Basis
Best fit
Coefficients
Consensus
NA
Factorization rank
C
CMS2_NTP
CMS2_SSP
CMS2_RF
CMS1_NTP
CMS1_SSP
CMS1_RF
CMS4_SSP
CMS4_NTP
CMS4_RF
CMS3_NTP
CMS3_RF
CMS3_SSP
-log10Pval
S-1
S-2
S-3
S-4
D
84
135
70
76
MSI
DNA repair
HNF4A
MSS
MYC
WNT
cell cycle
differentiation
glycolysis
fatty acids
CDX2
LGR5 stem-cells
TGF-Beta
EMT
dn
up
$-\log_{10}(p)$
S.1
S.2
S.3
S.4

Supplementary Figure 1. Evaluation of the NMF subtyping result.

A. Silhouette width plot of 365 colorectal cancer samples. Silhouette width measures the similarity between a sample and its corresponding cluster. A high value indicates that the sample is well matched. Each horizontal line represents a colorectal cancer sample and the length of the line is the silhouette width of sample. The average silhouette width of each cluster and all samples are showed on the graph.

B. Evaluation indexes across different clustering solutions (from 2 to 6). Mainly based on and the cophenetic correlation coefficient and the average silhouette width, we considered cluster member = 4 to be the preferred solution.

C. Sample overlapping significance heatmap. The p-value means the overlap statistically significance between our subtyping result and the CMS system (Fisher's exact test). Columns represent four subtypes given by NMF, and rows represent four CMS subtypes predicted by three CMS classifiers (NTP: the nearest template predictor, RF: the random forest, SSP: the single sample predictor).

D. Functional characteristics of the four subtypes. We used the R package 'CMScaller' to perform gene set analysis and visualize the result. Each row is a gene set. Each column represents a cancer subtype. The top bar shows subtype sizes.

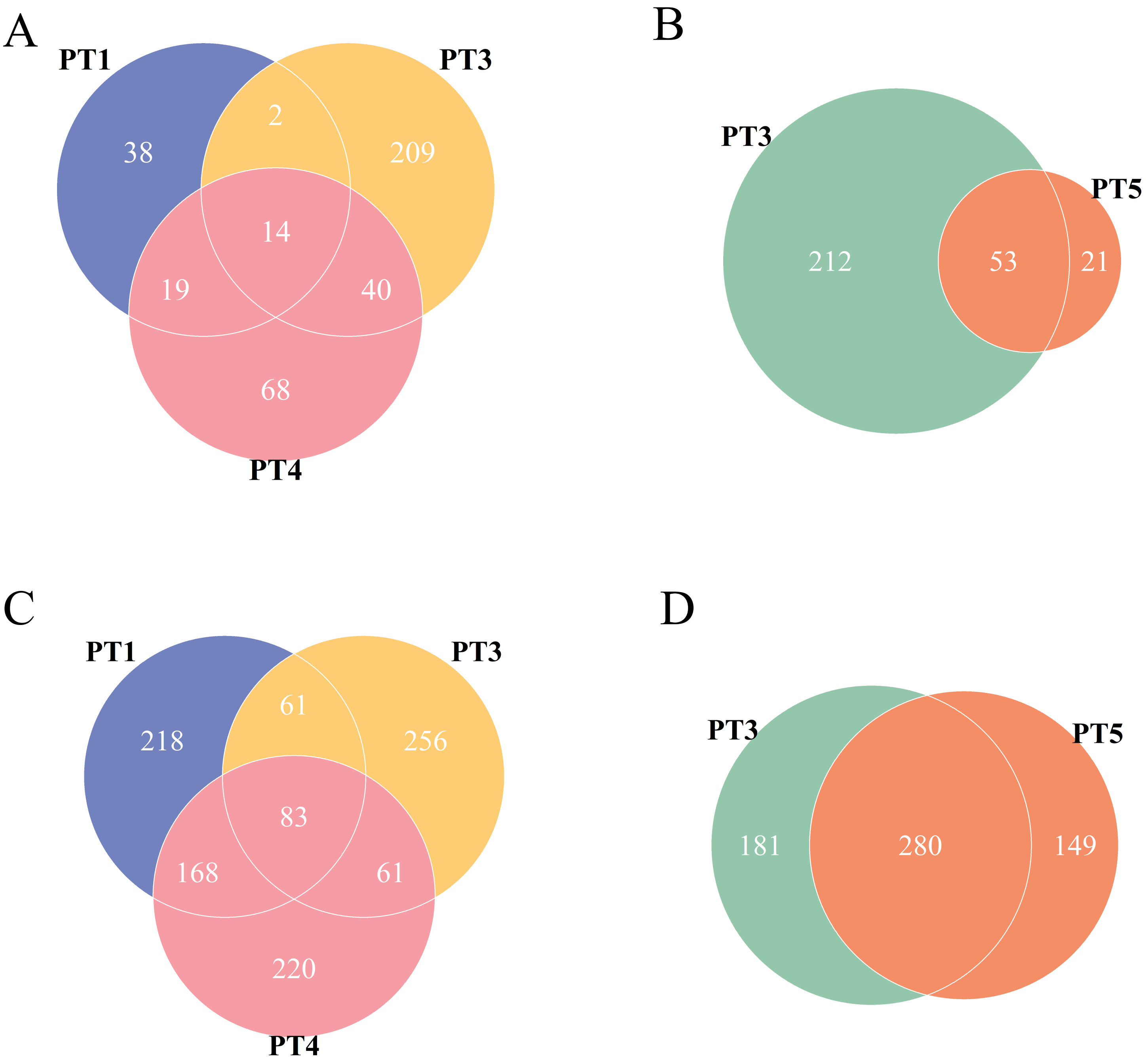
A
PT1
PT3
PT4
38
2
209
14
19
40
68
B
PT3
PT5
212
53
21
C
PT1
PT3
PT4
218
61
256
83
168
61
220
D
PT3
PT5
181
280
149

Supplementary Figure 2. The overlap between DEGs of the disease signatures of five colorectal patients.

A-B. Venn diagram summarizing S-4 (left) and S-3 (right) shared DEGs before purification.

C-D. Venn diagram summarizing S-4 (left) and S-3 (right) shared DEGs after purification.

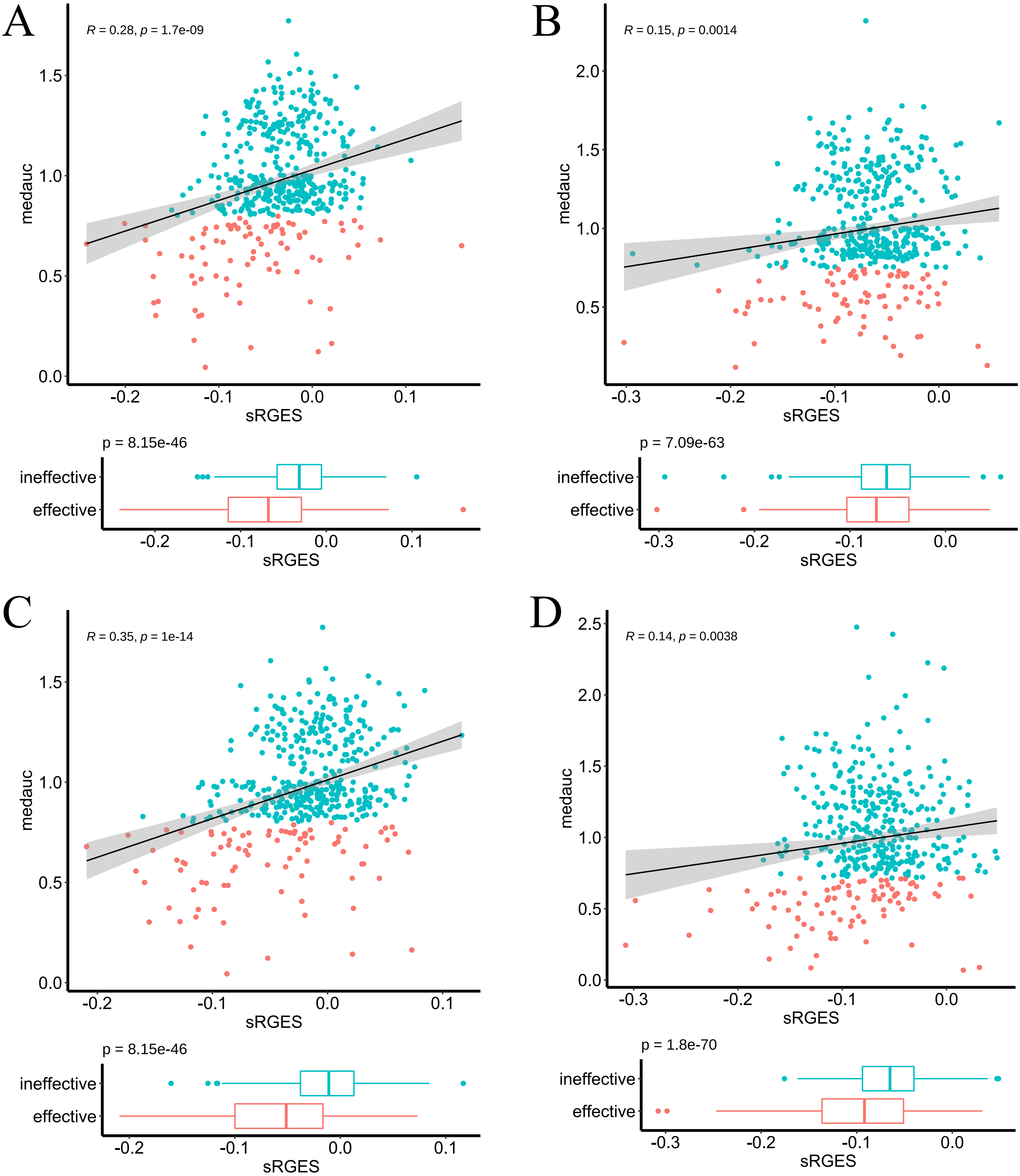

A
R = 0.28, p = 1.7e-09
medauc
sRGES
p = 8.15e-46
ineffective
effective
B
R = 0.15, p = 0.0014
medauc
sRGES
p = 7.09e-63
ineffective
effective
C
R = 0.35, p = 1e-14
medauc
sRGES
p = 8.15e-46
ineffective
effective
D
R = 0.14, p = 0.0038
medauc
sRGES
p = 1.8e-70
ineffective
effective

Supplementary Figure 3. In silico efficacy estimation drug prediction results for PT2 (A), PT3 (B), PT4 (C) and PT5 (D). The top panel shows the correlation analysis between sRGESs and efficacy AUCs of the predicted drugs, and the bottom panel shows sRGES differential analysis (t-test) between effective and ineffective drugs on patient-relevant cell line. The y-axis of the top panel represents the median AUC of multiple treatments of a drug on this cell line.
